# Partial mycoheterotrophy in the arbuscular mycorrhizal *Gentiana squarrosa* (Gentianaceae) demonstrated by coculture assays using C_3_ and C_4_ plants

**DOI:** 10.1007/s00572-026-01271-6

**Published:** 2026-05-28

**Authors:** Masahide Yamato, Moe Sasuga, Keito Shimabukuro, Ryota Kusakabe, Kenji Suetsugu

**Affiliations:** 1 Graduate School of Education, Chiba University, 1-33, Yayoi-cho, Inage-ku, Chiba, 263- 8522 Japan; 2 Faculty of Education, Chiba University, 1-33, Yayoi-cho, Inage-ku, Chiba, 263-8522 Japan; 3 Graduate School of Horticulture, Chiba University, 648, Matsudo, Matsudo, Chiba, 271- 8510 Japan; 4 Department of Biology, Graduate School of Science, Kobe University, Kobe, Hyogo 657- 8501 Japan

**Keywords:** Glomeromycotina, Hyphal network, Mixotrophy, Mycoheterotrophy, Stable isotope

## Abstract

Partial mycoheterotrophy is a nutritional mode in which green plants supplement photosynthetically derived carbon with fungal carbon obtained from associated fungi. In green arbuscular mycorrhizal (AM) plants, however, detecting partial mycoheterotrophy using natural ¹³C abundance is challenging, because the δ¹³C signatures of AM fungi often resemble those of their host plants. To overcome this limitation, we exploited the large natural δ¹³C contrast between C₃ and C₄ plants as an intrinsic tracer and tested for the incorporation of donor-derived carbon into the shoots of *Gentiana squarrosa* (Gentianaceae). Using an AM fungus commonly associated with *G. squarrosa*, we conducted coculture experiments with either a C₃ or C₄ companion plant. A compartmentalized U-shaped coculture system was employed, in which the roots of the companion plant were separated from *G. squarrosa* seedlings by a nylon mesh that permitted only hyphal passage, thereby minimizing alternative carbon transfer pathways, such as indirect transfer via CO₂ released by donor root respiration. Shoot δ¹³C values of *G. squarrosa* were significantly higher when seedlings were connected to a C₄ companion than when connected to a C₃ companion. Furthermore, among individuals grown with a C₄ companion, shoot dry mass was strongly and positively correlated with shoot δ¹³C values. These results demonstrate the incorporation of donor-derived carbon into *G. squarrosa* via AM fungal networks, providing robust evidence of partial mycoheterotrophy and suggesting that the mycoheterotrophy contributed to growth in this AM-associated species.

**Supplementary Information:**

The online version contains supplementary material available at 10.1007/s00572-026-01271-6.

## Introduction

In mycorrhizal symbioses, colonization of different host plants by the same fungus can result in the formation of hyphal connections between host roots (Newman 1988). These hyphal connections can develop into fungal networks, and some achlorophyllous plants are known to obtain carbon and nitrogen from these fungi. Reliance on fungus-derived carbon and nitrogen, termed mycoheterotrophy (Leake 1994), has evolved in some forest understory plants in Orchidaceae, Ericaceae, Burmanniaceae, Triuridaceae, Gentianaceae, Petrosaviaceae, and other families (Merckx et al. 2013a). Dikarya fungi (Basidiomycota and Ascomycota) are the mycobionts of the mycoheterotrophic plants in the Orchidaceae and Ericaceae (Waterman et al. 2013). Due to the enrichment of ^13^C and ^15^N isotopes in these fungi, some green plants exhibiting higher levels of these heavy isotopes are suggested to be partially mycoheterotrophic (Gebauer and Meyer 2003; Bidartondo et al. 2004; Julou et al. 2005).

Most terrestrial plants form arbuscular mycorrhizas (AM) with Glomeromycotina fungi (Spatafora et al. 2016), and some mycoheterotrophic plants, such as Burmanniaceae, Triuridaceae, Gentianaceae, and Petrosaviaceae species, have symbiotic relationships with specific lineages of AM fungi (Waterman et al. 2013). AM morphology is broadly divided into the *Arum* and *Paris* types (Gallaud 1905; Smith and Smith 1997), and all mycoheterotrophic AM plants form *Paris*-type AM (Imhof et al. 2013). Accordingly, some green *Paris*-type AM plants might be partially mycoheterotrophic as an intermediate stage in the evolution toward full mycoheterotrophy. Indeed, ^13^C enrichment has been reported in some mycoheterotrophic AM plants (Merckx et al. 2010; Bolin et al. 2017; Gomes et al. 2020), and relatively higher ^13^C levels have also been reported in some photosynthetic plants with *Paris*-type AMs growing on shady forest floors (Giesemann et al. 2020, 2021). However, a study on ^13^C levels in understory plants in broad-leaved deciduous forests found a significant correlation between ^13^C enrichment and AM colonization (%), but ^13^C abundance was not affected by the AM morphology (*Arum* vs. *Paris*; Murata-Kato et al. 2022). Such ambiguity reflects the low carbon isotope fractionation of AM fungi (Nakano et al. 1999), which might be attributable to the lipid acquisition from host plants as a carbon source in AM symbiosis (Luginbuehl et al. 2017; Kobayashi et al. 2018; Gomes et al. 2020; Klink et al. 2020). Accordingly, additional evidence is needed to test whether putative partial mycoheterotrophic plants are truly mycoheterotrophic.

Although laboratory or in situ CO_2_ labeling experiments can directly demonstrate carbon transfer through common mycorrhizal networks (Simard et al. 1997; Klein et al. 2016), these methods are labor-intensive and technically demanding, and they only capture short-term fluxes (Hynson et al. 2013). Consequently, these approaches have led to contrasting conclusions among studies, complicating interpretation (Karst et al. 2023; Robinson et al. 2024). Combining stable isotope data with additional evidence, however, represents a promising strategy for evaluating partial mycoheterotrophy. Because ^13^C enrichment is significantly higher in C_4_ plants than in C_3_ plants because of the different photosynthetic mechanisms involved (Edwards and Walker 1983), differences in δ^13^C values between C_3_ and C_4_ host plants can be reflected in AM fungal spores (Nakano et al. 1999; Courty et al. 2015). Accordingly, exploiting the distinct δ^13^C signatures of C_3_ and C_4_ plants might further clarify fungal carbon transfer within AM networks. If a putative partially mycoheterotrophic plant grows among C_4_ vegetation, any carbon obtained through shared AM fungi might carry the ^13^C signal of C_4_ hosts, which is markedly stronger than that of C_3_ plants (Bolin et al. 2017; Suetsugu et al. 2020a, b). Cultivating candidate species with both C_3_ and C_4_ plants and comparing δ^13^C values thus represents an effective strategy to assess partial mycoheterotrophy.

The Gentianaceae angiosperm family, which forms *Paris*-type AM, comprises 1750 species in 102 genera and 7 tribes (Stevens 2017). Within Gentianaceae, *Voyria* (tribe Voyrieae), *Voyriella* (tribe Saccifolieae), *Cotylanthera*, *Exacum*, *Exochaenium*, and *Sebaea* (tribe Exaceae) feature fully mycoheterotrophic species (Bidartondo et al. 2002; Merckx et al. 2013a, b). This independent development of mycoheterotrophy within Gentianaceae species suggests that preadaptation for mycoheterotrophy might have occurred early in the history of the family.

Partial mycoheterotrophy has been noted in some green Gentianaceae plants. Cameron and Bolin (2010) observed enrichment of ^13^C and ^15^N in *Obolaria virginica* L. and ^15^N enrichment in *Bartonia virginica* (L.) Britton, Sterns, & Poggenb. Suetsugu et al. (2020a) also detected ^13^C enrichment in *Pterygocalyx volubilis* Maxim. in a habitat with C_4_ plants. The ^13^C-enriched photosynthate produced by C_4_ plants could have contributed to higher δ^13^C values in the shoots of *P. volubilis*.

More recently, mycoheterotrophic underground seedling growth (initial mycoheterotrophy), which was previously observed only in Orchidaceae and some Ericaceae among seed plants, was reported for the photosynthetic plant *Gentiana zollingeri* Fawc. (Yamato et al. 2021). Because the shoot weight of *G. zollingeri* was positively correlated with shoot δ^13^C in this Gentianaceae plant (Yamato et al. 2023), mycoheterotrophy was strongly suggested to significantly influence the growth. Additionally, Suetsugu (2025) reported a negative correlation between ^13^C enrichment and the leaf ratio for this plant at the shoot growth stage, highlighting its capacity to adjust photosynthetic investment according to the degree of fungal dependence. However, fungal carbon obtained during the underground seedling stage can affect shoot δ^13^C; thus, quantitative evaluation of partial mycoheterotrophy might be difficult in this species.

*Gentiana squarrosa* Ledeb., a spring-flowering Gentianaceae species in the section Chondrophyllae, is a small green herbaceous plant reaching approximately 2–10 cm in height. The plant is distributed in sunny meadows in Japan, China, Northern India, Eastern Siberia, Mongolia, the Amur region, and the Korean Peninsula. During seed germination, *G. squarrosa* forms green cotyledons on the soil surface.

We first characterized the AM fungal community associated with *G. squarrosa* in two habitats, revealing that the dominant mycobiont was phylogenetically identical to the specific mycobiont of mycoheterotrophic seedlings of *G. zollingeri*. An AM fungus within this clade was previously isolated and identified as conspecific with or closely related to *Dominikia aurea* (Glomeraceae) (Kusakabe and Yamato 2023).

As higher ^13^C enrichment in C_4_ plants than in C_3_ plants can be reflected in their associated AM fungi (Nakano et al. 1999; Courty et al. 2015), we hypothesized that δ^13^C of *G. squarrosa* individuals cocultured with either *Medicago sativa* L. (C_3_) or *Panicum maximum* Jacq. (C_4_) in symbiosis with an AM fungus could serve as an indicator of partial mycoheterotrophy. Specifically, if carbon transfer occurs through AM fungal connections, δ^13^C should be higher in *G. squarrosa* grown with a C_4_ companion plant than in those grown with a C_3_ companion plant. We further predicted that δ^13^C values would be positively correlated with the shoot growth of *G. squarrosa*.

Two culture systems were used to test these hypotheses, in which the *Dominikia* sp. described above was used as the AM fungal inoculum. In the first experiment, *G. squarrosa* seedlings and companion plants (C_3_ or C_4_ plants) were grown together in mixed cultures within the same pot. In the second experiment, we used a U-shaped culture system that separated the root systems of companion plants and *G. squarrosa* seedlings using a nylon mesh, thereby reducing the influence of companion plant root respiration on carbon acquisition by *G. squarrosa*.

## Materials and methods

### Sampling

Flowering *G. squarrosa* individuals were collected at two meadow sites in Chiba Prefecture, Japan: Inzai City (35.79° N, 140.10° E) on April 19, 2019, and Shiroi City (35.80° N, 140.10° E) on May 5, 2022. At each site, five flowering individuals separated by more than 1 m were collected (GSI1–GSI5 from Inzai and GSS1–GSS5 from Shiroi). For each individual, a soil core (diameter, 5 cm; depth, 10 cm) was collected beneath the shoot using a stainless steel cylinder to obtain the root system.

### Molecular identification of colonizing arbuscular mycorrhizal fungi

Total DNA was extracted from the entire collected root system of each plant (approximately 30–50 mg fresh weight) using the DNeasy Plant Mini Kit (Qiagen, Hilden, Germany). Partial nuclear small subunit ribosomal RNA gene (SSU rDNA) sequences of AM fungi were amplified by polymerase chain reaction (PCR) using primers AMV4.5NF and AMDGR (Sato et al. 2005), with Nextera Transposase Adapters Read 1 and Read 2 (Illumina, San Diego, CA, USA) attached to the 5’ ends of AMV4.5NF and AMDGR, respectively.

The first PCR mixture contained 1 µL of DNA extract (5 ng µL^− 1^), 12.5 µL of 2× KAPA HiFi HotStart ReadyMix (Kapa Biosystems, Woburn, MA, USA), and 0.3 µM of each primer in a total volume of 25 µL. Using a Gene Atlas thermal cycler (Fukuoka, Japan), amplification was performed using the following program: 95 °C for 3 min; 35 cycles of 98 °C for 20 s, 60 °C for 15 s, and 72 °C for 15 s; and final extension at 72 °C for 5 min. PCR products were purified using AMPure XP (Beckman Coulter, Brea, CA, USA).

Purified amplicons were diluted to 5 ng µL^− 1^ and used as templates for the second PCR. The second PCR mixture contained 2 µL of template DNA, 6 µL of 2× KAPA HiFi HotStart ReadyMix, and 2 µL each of Index Primer 1 and Index Primer 2 from the Nextera XT Index Kit (Illumina). The amplification program was as follows: 95 °C for 30 s, followed by 12 cycles of 98 °C for 10 s, 55 °C for 30 s, and 72 °C for 30 s. Purified second PCR products were sequenced on the NovaSeq 6000 platform (v1.5 chemistry; 2 × 250 bp paired-end) by a commercial sequencing service (Rhelixa, Tokyo, Japan). Demultiplexed FASTQ files were deposited in the DDBJ sequence read archive (https://www.ddbj.nig.ac.jp/index-e.html) under the accession number PRJDB40125.

Sequence processing was performed in QIIME 2 v2024.10 (Bolyen et al. 2019). Paired-end sequences were denoised and filtered for chimeras using the DADA2 plugin (Callahan et al. 2016). The first 20 and 22 nucleotides of the 5’ end of the forward and reverse sequences, respectively, were trimmed. The 3’ ends of the forward and reverse sequences were truncated at position 130. For amplicon sequence variants (ASVs) made by the DADA2 plugin, taxonomy assignments were performed against SILVA 138 (Quast et al. 2013) by classify-sklearn in the feature-classifier plugin (Bokulich et al. 2018).

ASVs assigned to AM fungi were clustered into operational taxonomic units (OTUs) at a similarity threshold of 97% using cluster-features-de-novo in the VSEARCH plugin (Rognes et al. 2016). After removing OTUs with low read counts (< 10 reads in total), the read counts of the OTUs were rarefied to 23,952 per sample using the “rrarefy” function in the vegan package 2.7.2 (Oksanen et al. 2017) of R version 4.5.2 (R Core Team 2025). After rarefaction analysis, the remaining 50 OTUs were sequentially numbered according to the total read counts.

A heatmap of the top 20 OTUs (each comprising > 1.0% of total reads) was generated using plot_heatmap in phyloseq 1.54.0 (McMurdie and Holmes 2013). Further, permutational multivariate analysis of variance (PERMANOVA; 1000 permutations) using the “adonis2” function of the vegan package was performed on the basis of the Bray–Curtis index to analyze differences in OTU composition between the Inzai and Shiroi sampling sites.

Representative sequences of the 20 OTUs (> 1.0% of total reads) were assigned to MaarjAM virtual taxa using BLAST+ (Camacho et al. 2009) against the MaarjAM database (Öpik et al. 2010) using the following criteria: identity ≥ 97%, query coverage ≥ 80%, and E-value <1e − 50. Representative sequences were deposited in the International Nucleotide Sequence Database Collaboration (INSDC) database under the accession numbers TADJ01000001–TADJ01000020.

To clarify the phylogenetic positions of the 20 OTUs, the SSU rDNA sequences of the described AM fungal species closely related to each representative OTU sequence were downloaded from INSDC database alongside representative sequences from Glomeromycotina families (Acaulosporaceae, Diversisporaceae, Entrophosporaceae, Gigasporaceae, Glomeraceae, Pacisporaceae, and Paraglomeraceae). Multiple sequence alignment was performed using MAFFT v7.525 (Katoh and Standley 2013). The best-fit substitution model was selected according to the corrected Akaike Information Criterion (Sugiura 1978) using ModelTest-NG v0.1.7 (Darriba et al. 2020). Maximum likelihood phylogenetic inference was conducted using RAxML-NG v1.2.0 (Kozlov et al. 2019) with 1000 bootstrap replicates (Felsenstein 1985). The phylogenetic tree was visualized using FigTree v1.4.4 (http://tree.bio.ed.ac.uk/software/figtree/).

### Pot experiment 1

In this pot experiment, *G. squarrosa* was cocultured with *M. sativa* (C_3_ plant) or *P. maximum* (C_4_ plant) as a companion plant in the same pot. An AM fungus (CI1701), identified as conspecific with or closely related to *D. aurea* (Kusakabe and Yamato 2023), was used as the inoculum, because it matched the most dominant mycobiont of *G. squarrosa* in this study.

This AM fungus was propagated in a pot using *M. sativa* as the host plant, and a small soil core (10 mm in diameter and 20 mm in length) containing colonized *M. sativa* roots was used as the inoculum. The inoculum core was buried in a pot containing 1 L of autoclaved soil medium consisting of Akadama soil (volcanic soil granules) and river sand (1:1, v: v). Seeds of *M. sativa* and *P. maximum* were sterilized using sodium hypochlorite with 1% effective chlorine, and seeds were sown onto the soil medium in the pots. Three pots were established for each plant species on March 13, 2021 (aM1–aM3 for *M. sativa* and aP1–aP3 for *P. maximum*). The pot cultures were set on a cultivation shelf MLR-1546 (Sanyo, Osaka, Japan) under a 16-h photoperiod with a photon flux density of approximately 40 µmol m^−2^s^− 1^ at approximately 25 °C. The seedlings were thinned to three per pot, and the pot cultures were fertilized by pouring 500 mL of Peters liquid fertilizer (25-5-20) at a nitrogen concentration of 100 mg L^− 1^ every 3 months. At the time of fertilization, shoots were cut because of the limited cultivation space on the shelf.

Seeds of *G. squarrosa* were collected on May 23, 2021, from the Shiroi site, and approximately 200 seeds per pot were sown on May 25, 2021. On March 5, 2022, 284 days after sowing, shoots of *G. squarrosa* were harvested, as well as leaves of *M. sativa* and *P. maximum.* Newly expanded leaves of *M. sativa* and *P. maximum* were selected for sampling.

### Pot experiment 2

**Fig. 1:**
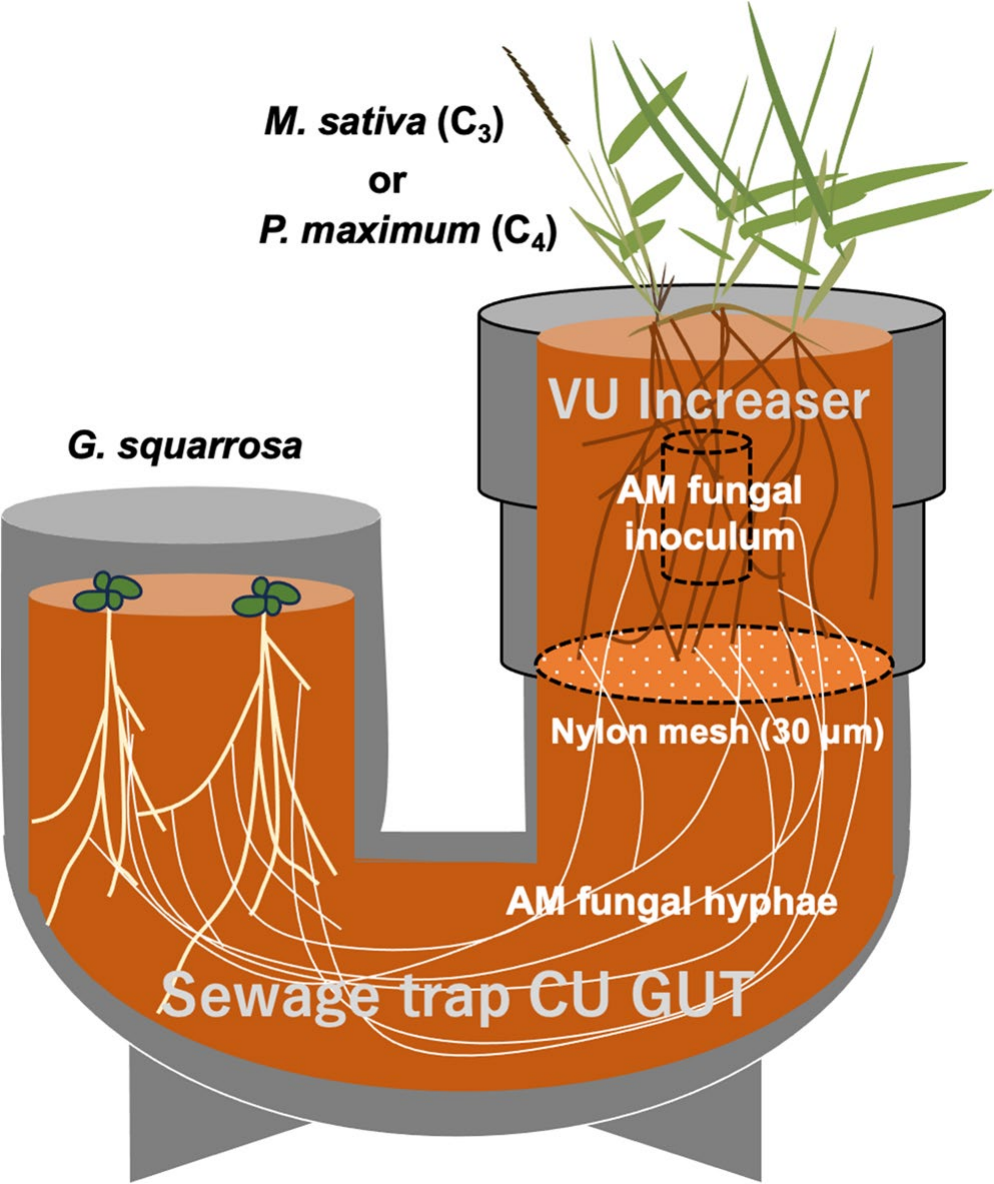
Diagram of the U-shaped culture system using a sewage trap CU GUT 50, VU Increaser 75 × 50, and nylon mesh with a 30-µm pore size. *Gentiana squarrosa* seedlings in the CU GUT compartment were grown with a companion plant, either *Medicago sativa* (C_3_ plant) or *Panicum maximum* (C_4_ plant). An arbuscular mycorrhizal fungus, *Dominikia* sp. CI1701, was inoculated into the VU Increaser compartment. The two compartments were separated by the nylon mesh

To minimize the potential effects of companion plant root respiration on the δ^13^C values of *G. squarrosa*, we devised a novel U-shaped culture system that separated the root system of *G. squarrosa* from the root compartment of the companion plant. Specifically, a VU Increaser 75 × 50 (Aronkasei, Tokyo, Japan) was connected to a sewage trap CU GUT 50 (Aronkasei) through a nylon mesh (30 μm pore size) to form the U-shaped culture system (Fig. 1). This pore size allowed hyphal passage while preventing root penetration.

On April 16, 2024, the culture system was filled with the aforementioned autoclaved soil medium and inoculated with *Dominikia* sp. CI1701 in the VU Increaser compartment as described for pot experiment 1. Surface-sterilized seeds of *M. sativa* or *P. maximum* were sown in the VU Increaser compartment. Simultaneously, seeds of *M. sativa* were sown in the CU GUT compartment to promote the AM fungal growth into this compartment. The U-shaped cultures were set on the cultivation shelf, and the seedlings were thinned into three in the VU Increaser compartment and one in the CU GUT compartment. The cultures were fertilized with 100 mL of Peters liquid fertilizer (25-5-20) at a nitrogen concentration of 100 mg L^− 1^, and then 50 mL of the fertilizer was added every month.

On August 2, 2024, small roots of *M. sativa* were collected from the CU GUT compartment using a cork borer, and AM fungal colonization was confirmed after staining the roots with 0.1% trypan blue. On September 17, 2024, growth improvement of *M. sativa* was confirmed in all cultures, indicating the successful induction of the AM fungus into the compartment. Then, approximately 200 seeds of *G. squarrosa*, collected on May 25, 2023, in the Shiroi habitat, were sown in the CU GUT compartment. After confirming that *G. squarrosa* formed two leaves in addition to the cotyledons in November 2024, the shoot of *M. sativa* in the CU GUT compartment was removed. Further growth of *G. squarrosa* was induced in the cultures until harvest on April 20, 2025, 218 days after sowing.

### Morphological observation of the mycorrhizal roots of *Gentiana squarrosa*

In pot experiment 2, the entire collected root system of *G. squarrosa* was fixed and maintained in 70% ethanol. The roots were autoclaved at 121 °C for 20 min in 10% KOH and stained with 0.1% trypan blue or 0.1% chlorazol black E according to Brundrett et al. (1996). Stained roots were squashed on a microscope slide using a coverslip and then observed under a BX51 microscope equipped with differential interference contrast optics (Olympus, Tokyo, Japan). The AM fungal colonization in the cortical cells of the root was photographed using a DP72 CCD camera (Olympus), and AM fungal colonization rates were analyzed for the total collected roots using the intersection method with a 1-mm grid according to Giovannetti and Mosse (1980).

### Stable isotope analysis

The harvested shoots of *G. squarrosa* and leaves of *M. sativa* and *P. maximum* were dried at 60 °C for 48 h, and the dry weight of the shoots was measured. The dried materials were cut into fine pieces using small scissors in a small glass bottle.

The abundance of stable carbon and nitrogen isotopes was measured using Flash EA 1112-ConFlo IV-Delta V Advantage (Thermo Fisher Scientific, Waltham, MA, USA) at the Research Institute for Humanity and Nature (Kyoto, Japan). Stable carbon isotope ratios were measured for the samples of pot experiment 1, whereas stable carbon and nitrogen isotopes were measured for the samples of pot experiment 2.

The relative abundance of the stable isotopes was calculated as follows: δ^13^C or δ^15^N = (R_sample_/R_standard_−1) × 1000 (‰), where R_sample_ represents the ^13^C/^12^ or ^15^ N/^14^N ratio of the sample, and R_standard_ represents the ^13^C/^12^C ratio of Vienna Pee Dee Belemnite or the ^15^N/^14^N ratio of atmospheric N_2_. The isotope ratios were calibrated using laboratory standards: CERKU-03 (glycine, δ^13^C = − 34.92‰, δ^15^*N* = 2.18‰), CERKU-05 (l-threonine, δ^13^C = − 9.45‰), and CERKU-10 (l-alanine, δ^15^*N* = − 5.22‰), which are traceable back to the international standards (Tayasu et al. 2011). For the samples of pot experiment 1, the analytical standard deviations (SDs) of δ^13^C were 0.0370‰ for CERKU-03 and 0.0543% for CERKU-05. For the samples of pot experiment 2, the analytical SDs of δ^13^C were 0.0516‰ for CERKU-03 and 0.0838‰ for CERKU-05, and those of δ^15^N were 0.0726‰ for CERKU-03 and 0.0947‰ for CERKU-10.

### Statistical analysis

Regarding the shoot dry weight of *G. squarrosa*, the AM fungal colonization rate, and δ^13^C and δ^15^N values, after assessing the normality of the distribution using the Shapiro–Wilk test, the means were analyzed using a linear mixed-effects model fitted with companion plant species as a fixed effect (*M. sativa* and *P. maximum*) and pot as a random intercept to account for variation among pots by the lmer function (lme4 package 1.1–38 in R). The significance of the fixed effects was then assessed by t-test with Satterthwaite’s approximation for degrees of freedom.

Concerning the shoot dry weight of *G. squarrosa*, correlations with δ^13^C, δ^15^N, and AM fungal colonization rates were examined using Pearson’s test within each companion plant treatment, with significance determined by t-test.

## Results

### Molecular identification of arbuscular mycorrhizal fungi

**Fig. 2:**
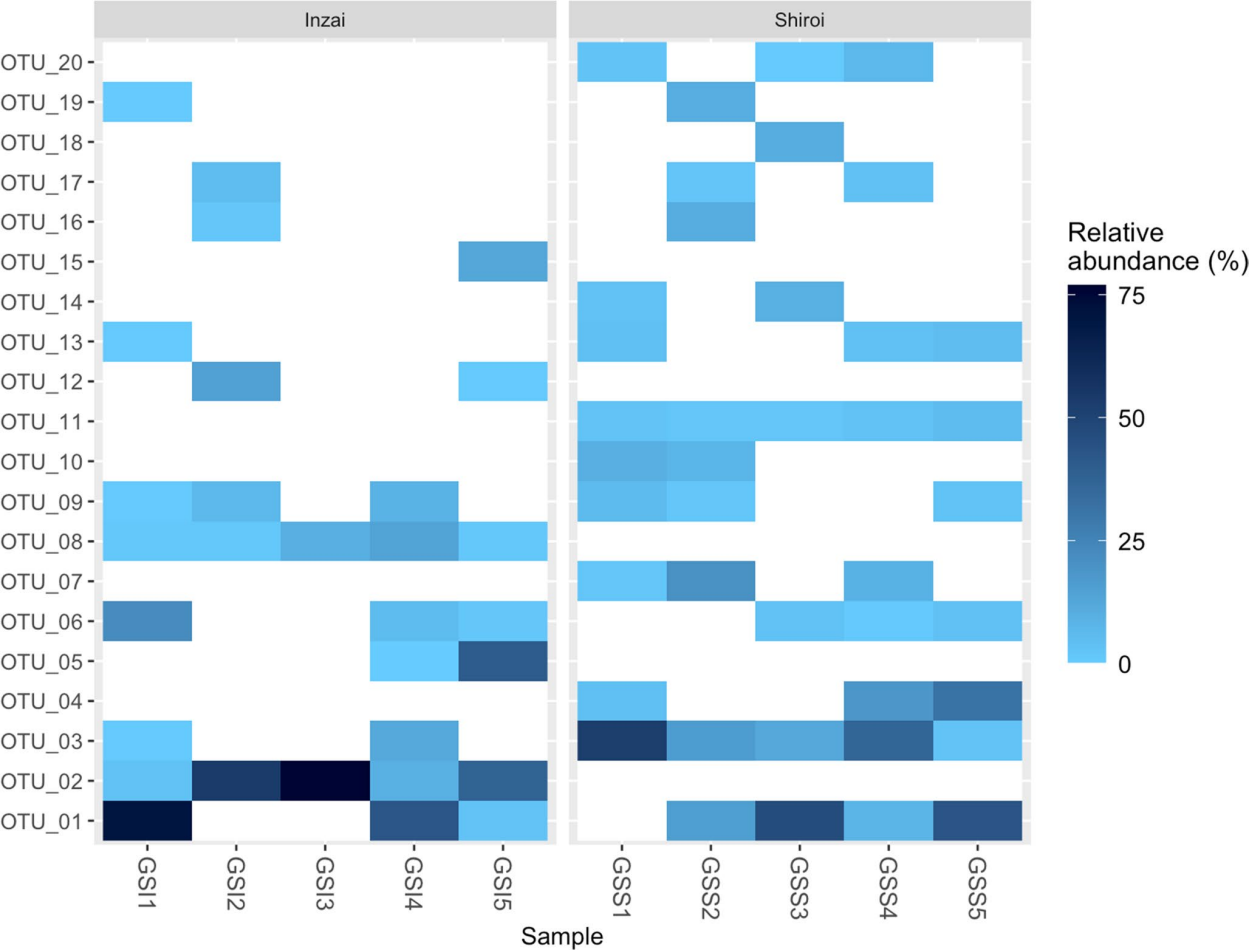
Heatmap of arbuscular mycorrhizal fungal operational taxonomic units (OTUs) based on relative abundance (%) estimated by read counts within each individual of *Gentiana squarrosa* collected from Inzai (GSI1-5) and Shiroi (GSS1-5). The 20 major OTUs (each representing > 1.0% of the total reads) are presented

Illumina sequencing yielded 391,226 high-quality AM fungal DNA sequences. After removing rare OTUs with fewer than 10 sequences and performing rarefaction, 239,520 reads were classified into 50 OTUs. Among them, 20 major OTUs comprising more than 1.0% of the total rarefied reads were selected, and their occurrence in the sampled plants is presented in Fig. 2. Furthermore, their assignments to VTXs and AM fungal families are presented in Table 1. The most dominant OTU (OTU 01) constituted 23.0% of the total rarefied reads. A significant difference in the AM fungal community between Inzai and Shiroi was confirmed by PERMANOVA based on the Bray–Curtis index (R^2^ = 0.298, F = 3.40, *p* = 0.0180).

**Table 1:** Allocation of the 20 most abundant OTUs (each representing >1.0% of the total reads) recovered from *Gentiana squarrosa* to MaarjAM virtual taxa (VTX; ≥97% sequence identity) and to arbuscular mycorrhizal fungal families (Glomeraceae [Glo], Entrophosporaceae [Ent], and Acaulosporaceae [Aca])

AM fungal OTUs																			
1	2	3	4	5	6	7	8	9	10	11	12	13	14	15	16	17	18	19	20
MaarjAM VTXs																			
166	83	345	397	393	159	NA	412	114	223	199	304	55	NA	188	276	193	360	NA	219
		108			186			387											
AM fungal families																			
Glo	Glo	Glo	Glo	Glo	Glo	Glo	Glo	Glo	Glo	Glo	Glo	Ent	Ent	Glo	Ent	Ent	Glo	Aca	Glo

*NA *No corresponding VTXs in MaarjAM (≥97% sequence identity)

The maximum likelihood phylogeny of representative sequences of the 20 AM fungal OTUs and selected species-identified AM fungi is presented in Fig. S1. OTU 01 formed a clade with a sequence of an AM fungus recently identified as conspecific or closely related to *D. aurea* (Kusakabe and Yamato 2023), which associates with mycoheterotrophic seedlings of *G. zollingeri* (Yamato et al. 2021).

### Pot experiment 1

In pot experiment 1, the results for the shoot dry weight, AM fungal colonization rate, and δ^13^C values of *G. squarrosa* grown with companion plants (*M. sativa* or *P. maximum*), along with δ^13^C values of the companion plants, are presented in Table S1. The germination rate of *G. squarrosa* was extremely low, and only one to three *G. squarrosa* plants were grown in each pot, resulting in the harvest of six and seven plants from cultures with *M. sativa* and with *P. maximum*, respectively.

For the companion plants, δ^13^C of *M. sativa* was − 34.91 ± 0.12‰ (mean ± SD), and that of *P. maximum* was − 14.59 ± 0.06‰. The shoot dry weights of *G. squarrosa* grown with *M. sativa* and *P. maximum* were 29.0 ± 12.3 mg and 33.9 ± 14.2 mg, respectively, and a linear mixed model analysis showed no significant effect of companion plants by t-test (t = − 0.664, *p* = 0.520), in which the variance attributed to the random effect of pot was negligible.

The δ^13^C values of *G. squarrosa* shoots grown with *M. sativa* and *P. maximum* were − 37.59 ± 0.21‰ and − 36.0 ± 0.43‰, respectively (Fig. S2). In a linear mixed model, variation among the pots was negligible, and the effect of companion plants was significant by t-test (t = − 8.27, *p* = 4.77 × 10^− 6^).

Among individuals grown with *P. maximum*, the shoot dry weight was significantly correlated with δ^13^C (Figure S3; *R* = 0.842, t = 3.49, *p* = 0.0176).

### Pot experiment 2

In pot experiment 2, the growth of companion plants (*M. sativa* and *P. maximum*) and *G. squarrosa* at sampling is presented in Fig. S4. One to five *G. squarrosa* plants were grown in each pot, and 7 and 13 plants were harvested from cultures with *M. sativa* and *P. maximum*, respectively. The shoot dry weight, AM fungal colonization rates, and δ^13^C and δ^15^N values of *G. squarrosa* in U-shaped cultures, along with δ^13^C and δ^15^N values of companion plants (*M. sativa* or *P. maximum*), are presented in Table S2.

**Fig. 3:**
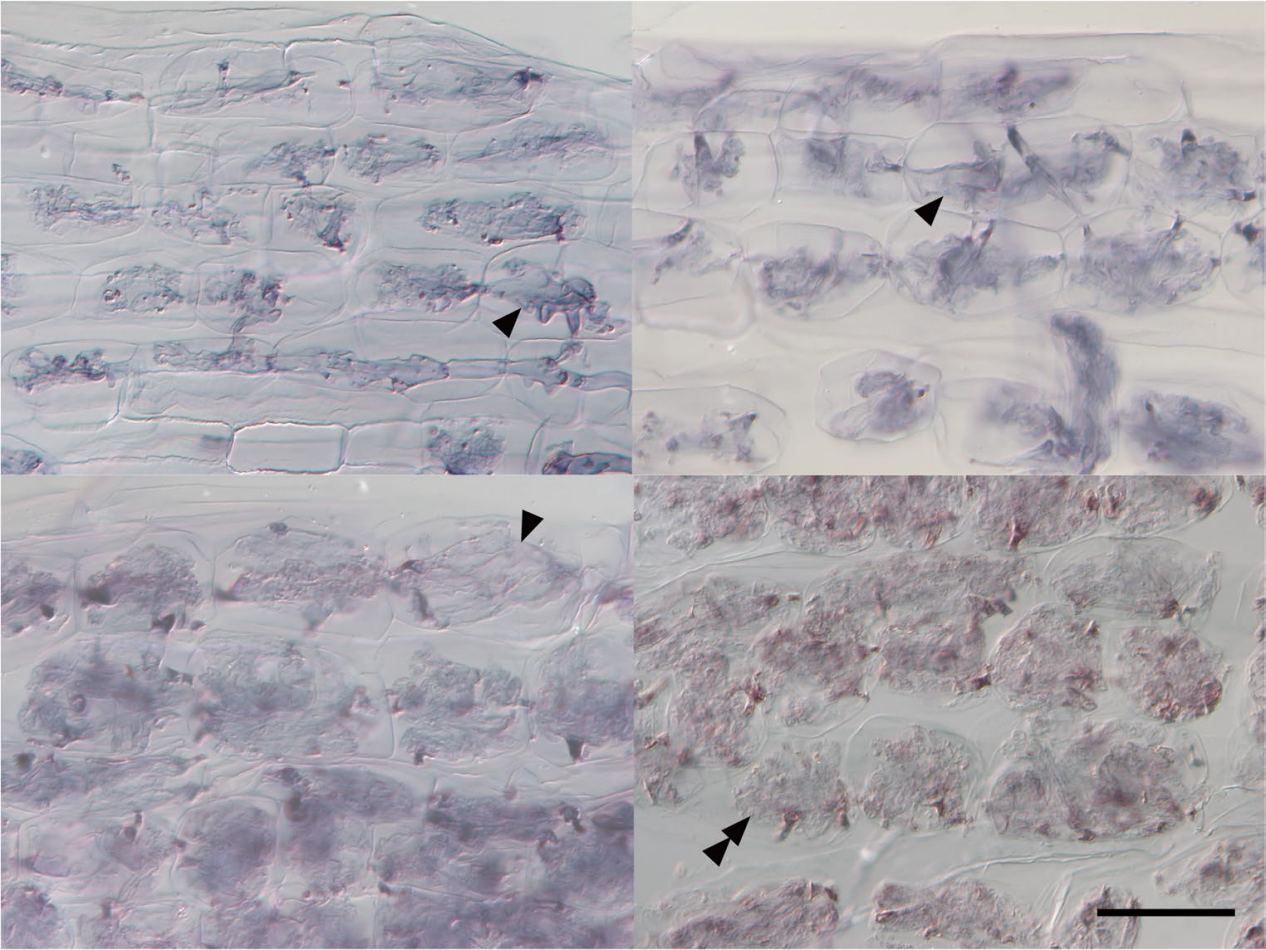
Hyphal coils of the arbuscular mycorrhizal fungus *Dominikia* sp. colonizing the root cortical cells of *Gentiana squarrosa*. *Arrowheads*: irregular hyphal swellings; *double arrowhead*: degenerated hypha. Bar: 50 μm

*Paris*-type AM structures with hyphal coils and hyphal degeneration were observed in cortical cells (Fig. 3). The colonization rates were 47.9 ± 8.2% with *M. sativa* and 44.0 ± 6.2% with *P. maximum*, and a linear mixed model analysis showed no significant effect of companion plants by t-test (t = − 1.20, *p* = 0.284), in which the variance attributed to the random effect of pot was 0.9552.

The shoot dry weights of *G. squarrosa* grown with *M. sativa* and *P. maximum* were 16.1 ± 6.48 mg and 12.4 ± 5.78 mg, respectively, and a linear mixed model analysis showed no significant effect of companion plants by t-test (t = − 1.31, *p* = 0.207), in which the variance attributed to the random effect of pot was negligible.

**Fig. 4:**
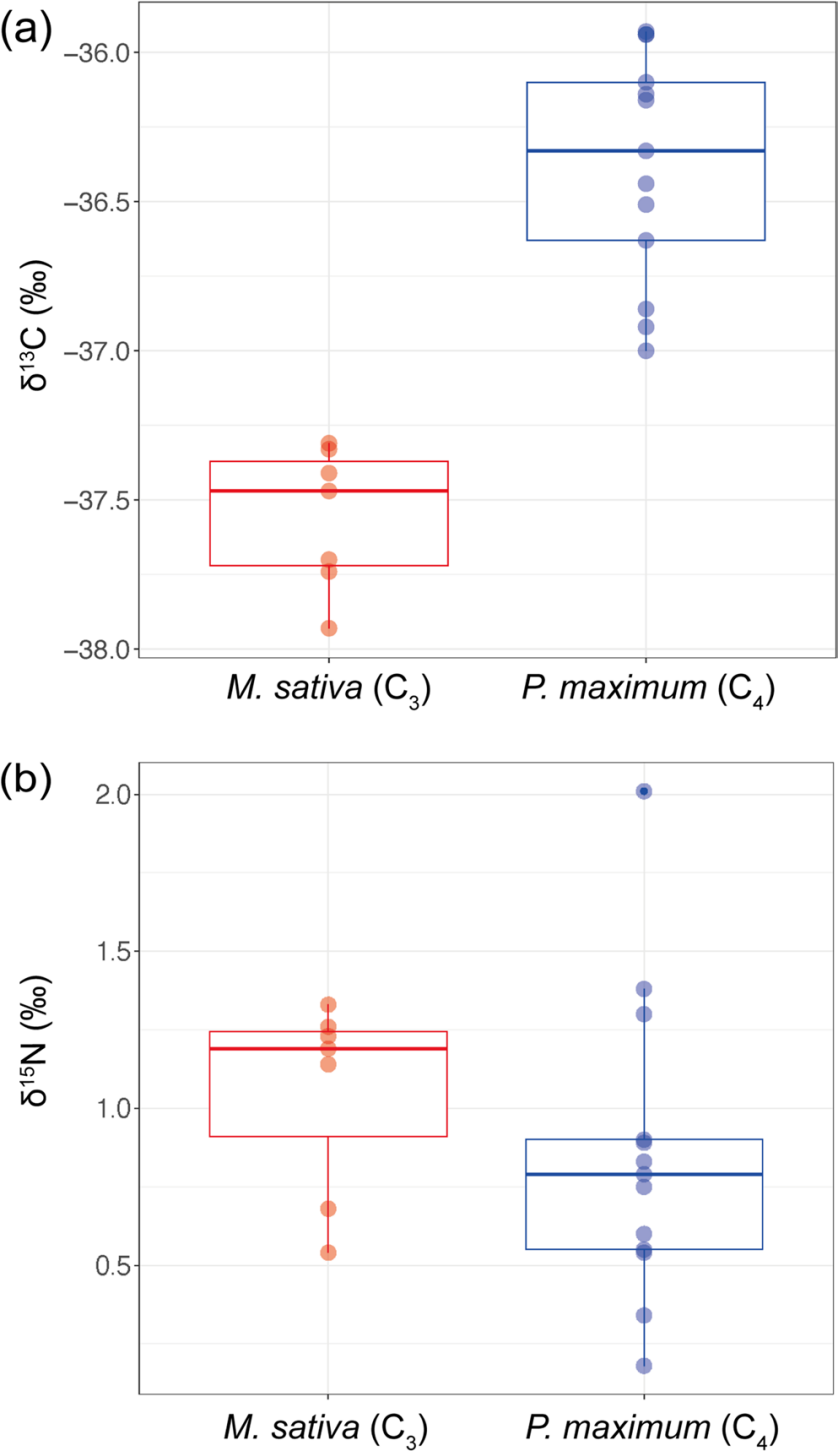
Boxplots of δ^13^C (**a**) and δ^15^N (**b**) of *Gentiana squarrosa* shoots grown with *Medicago sativa* (C_3_ plant) and *Panicum maximum* (C_4_ plant) in the U-shaped culture system (pot experiment 2)

For the companion plants, δ^13^C of *M. sativa* was − 35.76 ± 0.49‰, and that of *P. maximum* was − 16.15 ± 0.27‰. The δ^13^C values of *G. squarrosa* shoots grown with *M. sativa* and *P. maximum* were − 37.56 ± 0.31‰ and − 36.38 ± 0.85‰, respectively (Fig. 4a). In a linear mixed model, variation among the pots was negligible, and the effect of companion plants was significant by t-test (t = − 7.37, *p* = 7.79 × 10^− 7^).

**Fig. 5:**
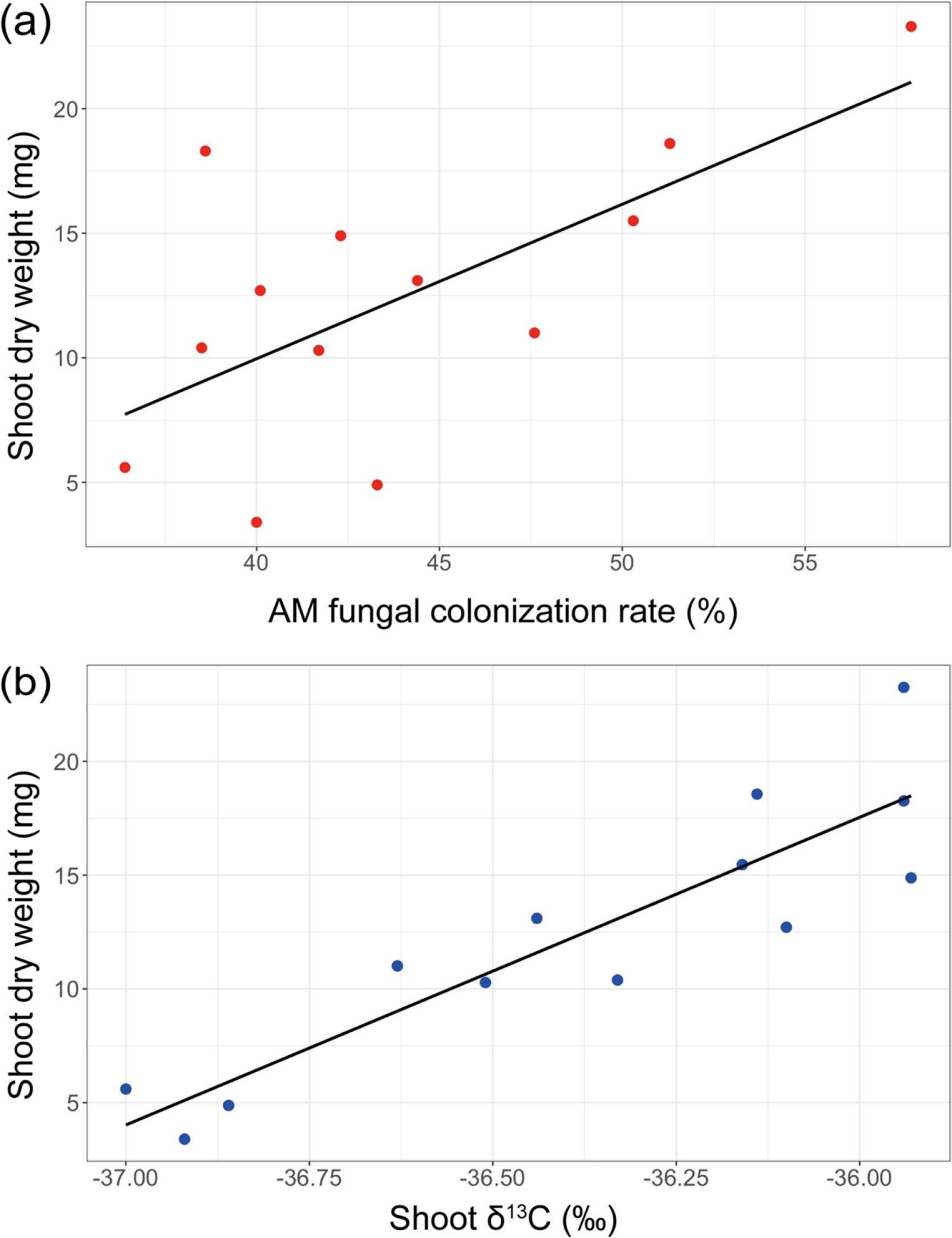
Relationships between (**a**) arbuscular mycorrhizal fungal colonization rate and shoot dry weight, and (**b**) shoot δ^13^C values and shoot dry weight, for *Gentiana squarrosa* grown with *Panicum maximum* (C_4_ plant) in the U-shaped culture system (pot experiment 2)

In the culture with *P. maximum* as the companion plant, the *G. squarrosa* shoot dry weight was significantly correlated with the AM fungal colonization rate (*R* = 0.660, t = 2.92, *p* = 0.014; Fig. 5a) and strongly correlated with δ^13^C (*R* = 0.898, t = 6.75, *p* = 3.14 × 10^− 5^; Fig. 5b). In contrast, for the culture with *M. sativa* as the companion plant, no significant correlations were found between *G. squarrosa* shoot dry weight and AM fungal colonization rate or δ^13^C values (Table S3).

For the nitrogen isotopes of companion plants, δ^15^N of *M. sativa* was − 1.04 ± 0.26‰, and that of *P. maximum* was − 0.86 ± 0.12‰. The δ^15^N values of *G. squarrosa* shoots grown with *M. sativa* and *P. maximum* were 1.05 ± 0.31‰ and 0.86 ± 0.48‰, respectively (Fig. 4b). A linear mixed model analysis showed no significant effect of companion plants on δ^15^N values by t-test (t = − 0.314, *p* = 0.764), in which the variance attributed to the random effect of pot was 0.097. Although δ^15^N values of *G. squarrosa* were relatively higher than those of companion plants, *M. sativa* and *P. maximum* (Table S2), no significant correlations were found between *G. squarrosa* shoot dry weight and δ^15^N values in both companion plant treatments (Table S3).

## Discussion

### Arbuscular mycorrhizal fungi associated with *G. squarrosa*

Surveys of AM fungal communities at two sites indicated limited specificity of *G. squarrosa* to a single narrow AM lineage. Phylogenetic analysis showed that the most dominant OTU clustered with an AM fungus identified as conspecific with or closely related to *D. aurea* (Kusakabe and Yamato 2023). This clade includes the mycobiont of mycoheterotrophic seedlings of *G. zollingeri* (Yamato et al. 2021) and corresponds to VTX166 in MaarjAM (Öpik et al. 2010). VTX166 is common in Japanese temperate forests, and it has been observed in both forest and grassland ecosystems (Miyake et al. 2020; Djotan et al. 2022; Větrovský et al. 2023; Kusakabe et al. 2024), suggesting that it represents a widespread dominant AM fungal lineage. According to MaarjAM, VTX166 also includes mycobionts reported from some mycoheterotrophic plants, such as *Burmannia championii*, *B. nepalensis* (Burmanniaceae), *Gymnosiphon divaricatus*, *Sciaphila ledermannii*, and *S. tosaensis* (Triuridaceae), suggesting that this lineage might be susceptible to exploitation by mycoheterotrophs (Perez-Lamarque et al. 2020). We therefore used strain CI1701 from this clade, which was isolated previously (Kusakabe and Yamato 2023), as the inoculum in the present pot experiments. Meanwhile, given the limited specificity observed in the field, other AM fungi might also be capable of sustaining partial mycoheterotrophy in *G. squarrosa*. This possibility warrants further study to determine whether only certain susceptible fungi can transfer carbon to partially mycoheterotrophic plants.

### The ^**13**^C data are consistent with partial mycoheterotrophy in *G. squarrosa*

Because of the lower carbon isotope fractionation of AM fungi (Nakano et al. 1999), we cocultured *G. squarrosa* with companion plants that differed strongly in δ^13^C: *M. sativa* (C_3_) and *P. maximum* (C_4_). In pot experiment 1, *G. squarrosa* was grown in mixed culture with each companion plant in the same pot. δ^13^C was significantly higher for *G. squarrosa* grown with *P. maximum* than for that grown with *M. sativa*, mirroring the large δ^13^C difference between the companion plants. This pattern suggested carbon acquisition by *G. squarrosa* from the companion plant through AM fungal connections. However, in a shared pot, CO_2_ derived from companion plant root respiration could also influence δ^13^C in *G. squarrosa*.

To reduce the potential effects of root respiration, pot experiment 2 used a U-shaped culture system in which the companion plant roots and the *G. squarrosa* compartment were separated by a 30-µm nylon mesh. In this system, the AM fungal colonization rate in *G. squarrosa* reached approximately 40%–50% in both treatments. Because the AM fungus was inoculated only into the companion plant compartment, these colonization levels indicate the successful establishment of hyphal connections between companion plants and *G. squarrosa*. The hyphal swellings and degenerating coils observed in the roots of *G. squarrosa* were consistent with reports from other Gentianaceae species (McGee 1985; Imhof 1999; Yamato et al. 2021), although further work is needed to clarify their functional significance.

In both experiments, δ^13^C was lower for *G. squarrosa* than for *M. sativa*, which might reflect species-specific variation in δ^13^C among C_3_ plants (Murata-Kato et al. 2022). Nevertheless, the difference in δ^13^C was again reflected in a significant difference in δ^13^C for *G. squarrosa* cocultured with *M. sativa* and *P. maximum*. Although *M. sativa* was transiently used to promote fungal induction into the *G. squarrosa* compartment, its influence on the treatment comparison is likely limited. This is because the same induction step was applied to both treatments, and the effect of the C_3_ promoter plant *M. sativa* on the δ^13^C of *G. squarrosa* (also a C_3_ plant) was expected to be minor, considering the large contrast between C_3_ and C_4_ plants.

The strong positive correlation between δ^13^C and shoot dry mass in *G. squarrosa* grown with *P. maximum* further suggests that donor-derived carbon acquisition contributed to growth under the experimental conditions. Collectively, these results support donor-derived carbon incorporation by *G. squarrosa* through AM fungal connections, consistent with partial mycoheterotrophy, particularly in the compartmentalized U-shaped culture system. The difference in δ^13^C values of *G. squarrosa* between the *M. sativa* and *P. maximum* treatments was 1.59‰ in pot experiment 1 and 1.18‰ in pot experiment 2. Although AM fungal colonization was not evaluated in pot experiment 1, the denser contact between *G. squarrosa* roots and the AM symbiosis of companion plants in the same pot, combined with the longer cultivation period, may have influenced this difference. While a control without companion plants would better clarify the role of AM networks in carbon acquisition, *G. squarrosa* showed poor growth without AM colonization in preliminary trials. Direct spore inoculation also failed to induce symbiosis in young seedlings because of low spore germination. Employing commercial AM fungal strains with higher germination rates could resolve these issues in future studies.

In mycoheterotrophic plants, nitrogen transfer from fungi can accompany carbon transfer (Gebauer and Meyer 2003; Bidartondo et al. 2004). In this study, δ^15^N did not differ between companion plants, limiting comparison between treatments. However, δ^15^N was higher in *G. squarrosa* than in the companion plants in both treatments, consistent with patterns reported for mycoheterotrophic AM plants (Gomes et al. 2020). Because δ^15^N was not correlated with shoot dry mass, further work is needed to evaluate fungal nitrogen acquisition and its contribution to growth.

Although partial mycoheterotrophy has been inferred from ^13^C and ^15^N enrichment in several green Gentianaceae species (Cameron and Bolin 2010; Suetsugu et al. 2020a; Suetsugu 2025), this study differs from earlier work in that it directly demonstrated a significant effect on plant growth. Due to the modest difference in δ^13^C values between treatments, the amount of fungal carbon acquired by *G. squarrosa* seedlings appears to be low. Nevertheless, the significant correlation between δ^13^C and shoot dry mass suggests the ecological importance of mycoheterotrophy, particularly during the early growth stages. As *G. squarrosa* typically inhabits sunny meadows, further research is needed to determine the actual contribution of mycoheterotrophy in its natural habitat. Many Gentianaceae plants grow in dark understories, where fungal carbon subsidies might be more important. Given the repeated origins of mycoheterotrophy and shared AM morphology within Gentianaceae plants, partial mycoheterotrophy might be more widespread in this family than currently recognized.

### Future studies on the autotrophy–mycoheterotrophy continuum

Some studies have proposed that resources are transferred among forest plants through common mycorrhizal networks, resulting in increased seedling performance (Read 1997; Simard et al. 1997). Although this idea has often been highlighted in popular media, robust evidence for large net transfers among green plants remains limited (Karst et al. 2023). Meanwhile, mycoheterotrophy demonstrates that carbon transfer can occur through mycorrhizal symbioses between green and nonphotosynthetic plants (Merckx et al. 2024). Partial mycoheterotrophy, in which green plants obtain part of their carbon from associated fungi, has been demonstrated in some Orchidaceae and Ericaceae plants (Gebauer and Meyer 2003; Bidartondo et al. 2004; Julou et al. 2005; Hynson et al. 2013), although these cases occur in relatively restricted ecological contexts. Because AM association is the most ubiquitous mycorrhizal type, the partial mycoheterotrophy and associated growth enhancement suggested in this study for *G. squarrosa* likely offer a broader perspective on carbon transfer through common mycorrhizal networks.

Across partially mycoheterotrophic plants, carbon acquisition from mycorrhizal fungi can vary over evolutionary, developmental, and ecological scales (Merckx et al. 2024). Along this autotrophy–mycoheterotrophy continuum, quantifying the degree of fungal carbon gain remains difficult for field-collected AM plants (Giesemann et al. 2020, 2021; Murata-Kato et al. 2022), as the carbon isotope fractionation is low in AM fungi (Nakano et al. 1999). Additionally, laboratory or in situ CO_2_ labeling experiments are labor-intensive and technically demanding, and they only capture short-term fluxes (Hynson et al. 2013). We therefore propose using our developed U-shaped culture system, a compartmentalized coculture design with C_3_ and C_4_ plants, to assess partial mycoheterotrophy among understory *Paris*-type AM plants under controlled conditions. We also note that even in this system, carbon acquisition by *G. squarrosa* from companion plant-derived CO_2_ might not be eliminated completely. However, the strong positive correlation between shoot δ^13^C and the shoot dry mass in *G. squarrosa* grown with C_4_ plants suggests a substantial contribution of the mycoheterotrophic pathway under the experimental conditions.

## Conclusion

We found evidence for partial mycoheterotrophy in *G. squarrosa* by coculturing seedlings with C_3_ or C_4_ companion plants that differed in δ^13^C and inoculating the cultures with an AM fungus that dominated field-collected *G. squarrosa* roots from two habitats. We used a U-shaped culture system with a nylon mesh to separate companion plant roots from *G. squarrosa* seedlings, thereby reducing the potential effects of companion plant root respiration on carbon acquisition by *G. squarrosa*. In this system, *G. squarrosa* shoots grown with C_4_ plants exhibited significantly higher δ^13^C than those grown with C_3_ plants, along with a positive correlation between the shoot δ^13^C and the shoot dry mass. This result supports the existence of partial mycoheterotrophy in *G. squarrosa* and suggests that it can contribute to growth, at least under the present experimental conditions. This finding, together with recent studies (e.g., Giesemann et al. 2020, 2021), challenges the prevailing view that, in AM symbioses, carbon moves unidirectionally from plant to fungus, with fully mycoheterotrophic plants as the only exception (e.g., Pfeffer et al. 2004).

## Supplementary Information

Supplementary Material 1.

## Data Availability

Sequence data were generated in FASTQ format for each index primer pair and deposited in the DDBJ sequence archive under accession number PRJDB40125. The representative sequences of each OTU were deposited in the DDBJ under the accession numbers TADJ01000001–TADJ 01000020.
