# Supplementary material for: Partial mycoheterotrophy in the arbuscular mycorrhizal Gentiana squarrosa (Gentianaceae) demonstrated by coculture assays using C3 and C4 plants
Source: Mycorrhiza. 2026 May 28;36(3):27. doi: 10.1007/s00572-026-01271-6 (PMC13219101; doi:10.1007/s00572-026-01271-6)
Supplement: Supplementary file 1 — Supplementary Material 1. [file 572_2026_1271_MOESM1_ESM.pdf]

Supplementary Information

Mycorrhiza

Partial mycoheterotrophy in the arbuscular mycorrhizal *Gentiana squarrosa* (Gentianaceae) demonstrated by coculture assay using C_3 and C_4 plants

Masahide Yamato*[1], Moe Sasuga[1], Keito Shimabukuro[2], Ryota Kusakabe[3], Kenji Suetsugu[4]

[1]Graduate School of Education, Chiba University, Chiba, Japan
[2]Faculty of Education, Chiba University, Chiba, Japan
[3]Graduate School of Horticulture, Chiba University, Chiba, Japan
[4]Department of Biology, Graduate School of Science, Kobe University, Kobe, Japan

*Corresponding author: Masahide Yamato: myamato@chiba-u.jp

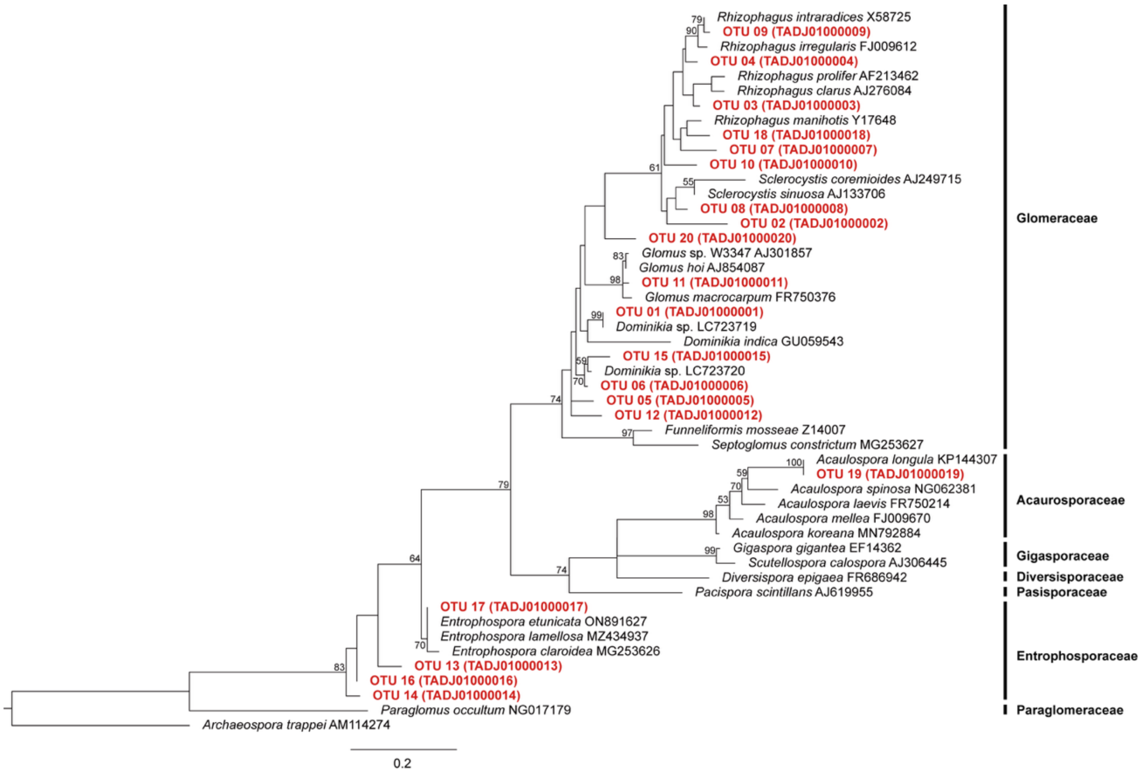

Fig. S1 Maximum likelihood phylogenetic tree based on the partial nuclear small subunit ribosomal RNA sequences of arbuscular mycorrhizal fungi. The tree comprises representative sequences from operational taxonomic units (OTUs) that account for >1.0% of the total rarefied reads obtained from *Gentiana squarrosa* in this study, along with sequences from the described species downloaded from GenBank. Accession numbers are provided for the obtained and downloaded sequences. The tree is rooted in *Archaeospora trappei* (AM114274). Bootstrap values ≥50% based on 1000 replicates are presented at each node. The scale bar represents the number of substitutions per site.

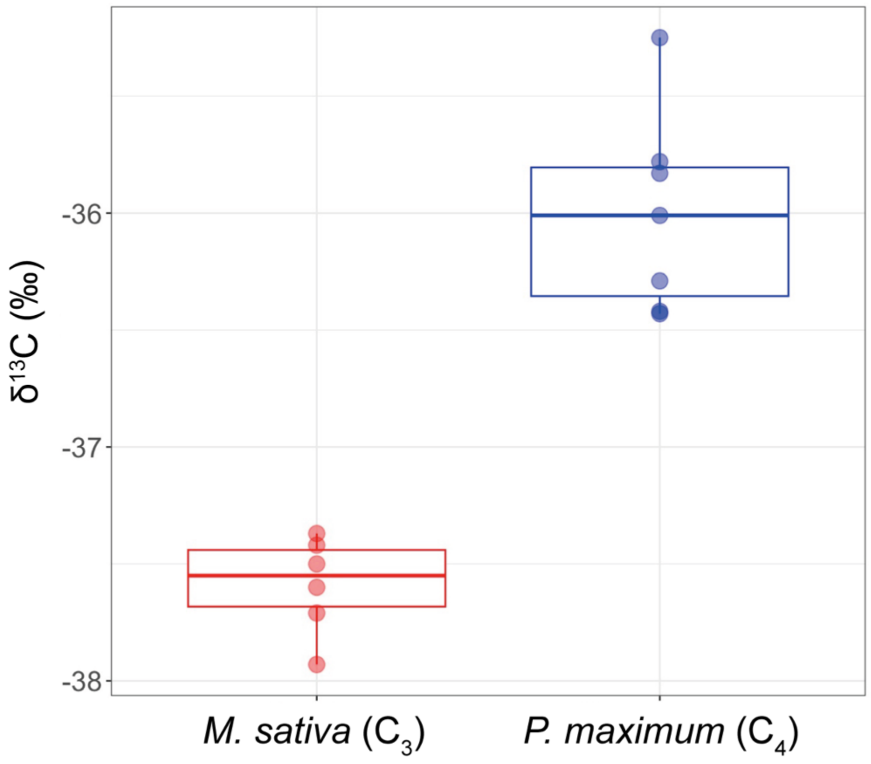

Fig. S2 Boxplots of $\delta^{13}C$ of *Gentiana squarrosa* shoots grown with *Medicago sativa* (C_3 plant) and *Panicum maximum* (C_4 plant) in the same pot (pot experiment 1).

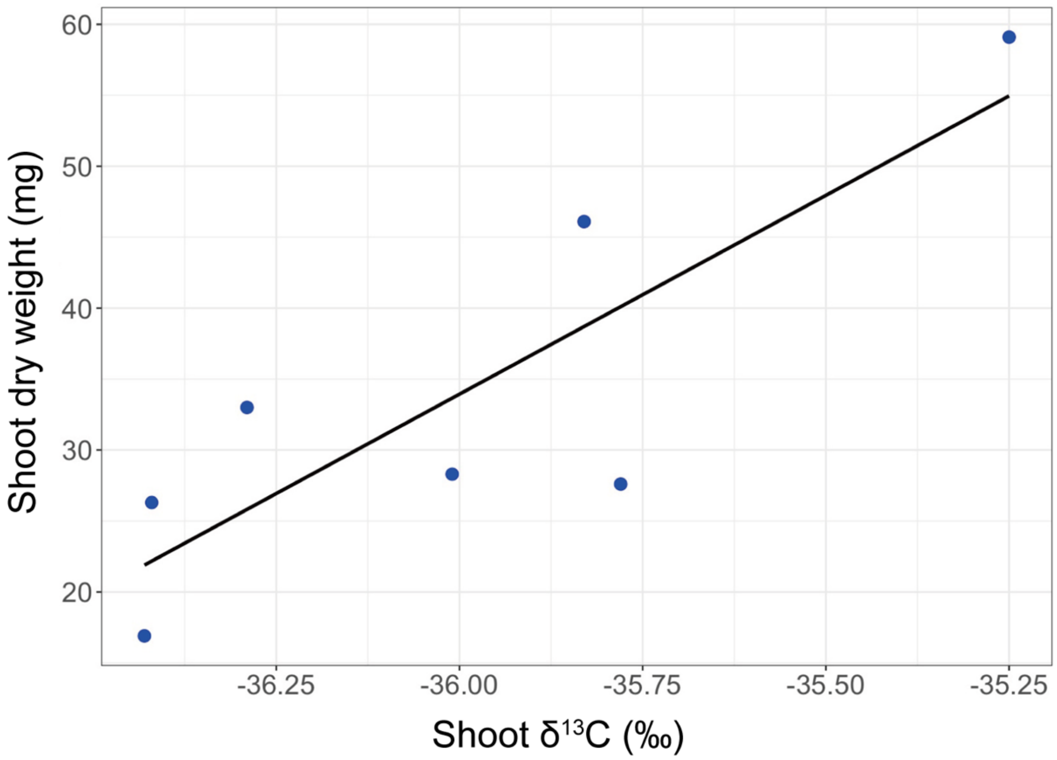

Fig. S3 Relationship between shoot $\delta^{13}C$ and the shoot dry weight of *Gentiana squarrosa* grown with *Panicum maximum* (C_4 plant) in the same pot (pot experiment 1).

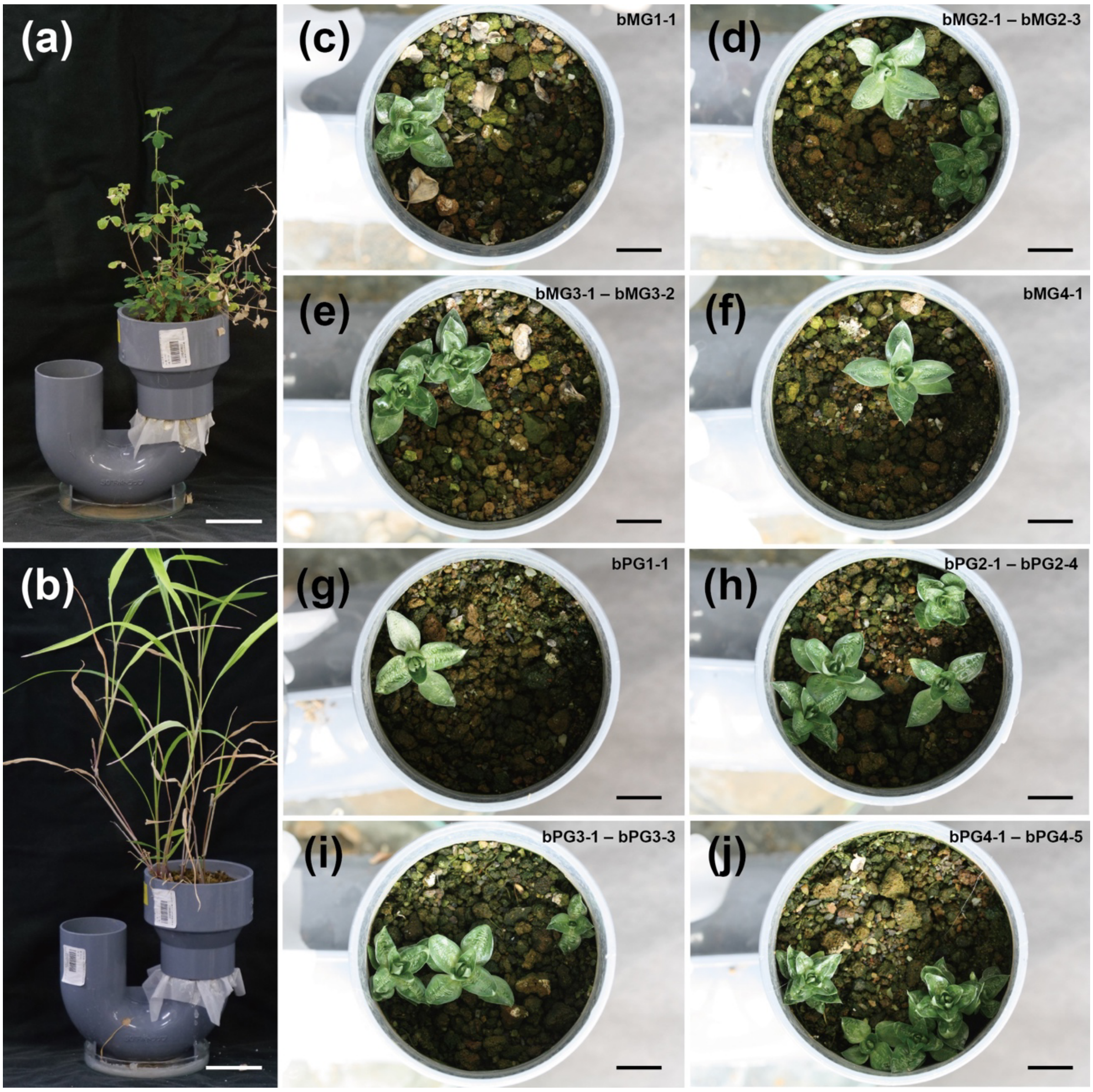

Fig. S4 Companion plants in the VU Increaser compartment, *Medicago sativa* (**a**) and *Panicum maximum* (**b**) and *Gentiana squarrosa* in the CU GUT (sewage trap) compartment, separated by a nylon mesh: cultures with *M. sativa* (**c**–**f**) and with *P. maximum* (**g**–**j**) at harvest (pot experiment 2). The sample code of *G. squarrosa* individuals (corresponding to Table S2) is shown in the upper right of each image. Scale bars: (**a**, **b**) 5 cm; (**c**–**j**) 1 cm.

Table S1 Shoot dry weight and $\delta^{13}C$ of *Gentiana squarrosa* in pot cultures grown with companion plants (*Medicago sativa* or *Panicum maximum*) along with $\delta^{13}C$ of the companion plants (pot experiment 1).

Companion plant			*G. squarrosa*		
Sample code	Species	$\delta^{13}C$ (‰)	Sample code	Shoot weight (mg)	$\delta^{13}C$ (‰)
aM1	*M. sativa*	-34.98	aMG1-1	44.6	-37.50
aM2	*M. sativa*	-34.77	aMG2-1	40.5	-37.42
			aMG2-2	28.2	-37.37
			aMG2-3	25.8	-37.60
aM3	*M. sativa*	-34.98	aMG3-1	24.6	-37.71
			aMG3-2	10.1	-37.93
aP1	*P. maximum*	-14.65	aPG1-1	28.3	-36.01
			aPG1-2	27.6	-35.78
aP2	*P. maximum*	-14.58	aPG2-1	59.1	-35.25
			aPG2-2	26.3	-36.42
			aPG2-3	16.9	-36.43
aP3	*P. maximum*	-14.53	aPG3-1	46.1	-35.83
			aPG3-2	33.0	-36.29

Table S2 Shoot dry weight, colonization rate of the arbuscular mycorrhizal fungus, $\delta^{13}C$, and $\delta^{15}N$ of *Gentiana squarrosa* cultured in U-shaped tubes grown with companion plants (*Medicago sativa* or *Panicum maximum*) along with $\delta^{13}C$ and $\delta^{15}N$ values of the companion plants (pot experiment 2).

Companion plant				*G. squarrosa*				
Sample code	Species	$\delta^{13}C$ (‰)	$\delta^{15}N$ (‰)	Sample code	Shoot weight (mg)	AM fungal colonization rate (%)	$\delta^{13}C$ (‰)	$\delta^{15}N$ (‰)
bM1	*M. sativa*	-35.28	-0.77	bMG1-1	18.8	61.1	-37.70	1.14
bM2	*M. sativa*	-35.8186	-0.89	bMG2-1	13.5	53.6	-37.31	1.23
				bMG2-2	13.0	50.5	-37.33	1.19
				bMG2-3	4.6	36.7	-37.93	0.68
bM3	*M. sativa*	-35.5249	-1.31	bMG3-1	21.5	42.8	-37.47	1.33
				bMG3-2	24.2	41.6	-37.41	1.26
bM4	*M. sativa*	-36.4264	-1.21	bMG4-1	17.5	49.2	-37.74	0.54
bP1	*P. maximum*	-16.2687	-0.72	bPG1-1	12.7	40.1	-36.10	1.30
bP2	*P. maximum*	-16.4379	-0.77	bPG2-1	14.9	42.3	-35.93	0.75
				bPG2-2	13.1	44.4	-36.44	0.89
				bPG2-3	10.3	41.7	-36.51	0.79
				bPG2-4	10.4	38.5	-36.33	0.34
bP3	*P. maximum*	-16.1087	-0.95	bPG3-1	18.3	38.6	-35.94	1.38
				bPG3-2	15.5	50.3	-36.16	2.01
				bPG3-3	3.4	40.0	-36.92	0.83
bP4	*P. maximum*	-15.7936	-0.98	bPG4-1	23.3	57.9	-35.94	0.54
				bPG4-2	18.6	51.3	-36.14	0.90
				bPG4-3	11.0	47.6	-36.63	0.60
				bPG4-4	5.6	36.4	-37.00	0.55
				bPG4-5	4.9	43.3	-36.86	0.18

Table S3 Pearson's correlation coefficients between shoot dry weight of *Gentiana squarrosa* and the arbuscular mycorrhizal fungal colonization rate (%), shoot $\delta^{13}C$ (‰) and $\delta^{15}N$, along with their p-values in parentheses, in U-shaped cultures grown with *Medicago sativa* (C_3 plant) or *Panicum maximum* (C_4 plant) in pot experiment 2.

Companion plant	AM fungal colonization rate (%)	$\delta^{13}C$ (‰)	$\delta^{15}N$ (‰)
M. sativa	0.202	0.431	0.504
n = 7	(0.663)	(0.335)	(0.249)
P. maximum	**0.660**	**0.898**	0.367
n = 13	**(0.014)**	**(3.14 x 10^{-5})**	(0.218)
